# Discussing Sexual Health with Adolescent and Young Adults with Cancer: a Qualitative Study Among Healthcare Providers

**DOI:** 10.1007/s13187-020-01796-0

**Published:** 2020-06-17

**Authors:** Leonore F. Albers, Folkertje B. Bergsma, Hilda Mekelenkamp, Rob C.M. Pelger, Eveliene Manten-Horst, Henk W. Elzevier

**Affiliations:** 1 Department of Urology, Leiden University Medical Centre, PO-box 9600, 2300 WB Leiden, The Netherlands; 2 Department of Medical Decision Making, Leiden University Medical Centre, PO-box 9600, 2300 WB Leiden, The Netherlands; 3 Department of Paediatric, Leiden University Medical Centre, PO-box 9600, 2300 WB Leiden, The Netherlands; 4 Dutch AYA “Young and Cancer” Platform, Godebaldkwartier 419, 3511 DT Utrecht, The Netherlands

**Keywords:** Sexual health, Adolescent and young adult (AYA), Cancer, Information provision, Healthcare provider (HCP)

## Abstract

Sexual health is an important aspect of quality-of-life for adolescent and young adult (AYA) patients with cancer. AYAs report a need for information about sexual health but experience a lack of communication with their healthcare provider. It is known that sexual health is a difficult theme for healthcare providers to discuss. This study aims to gain insight into perspectives of healthcare providers in facilitating AYAs’ needs regarding sexual health. A qualitative study was performed. Six doctors and eight nurses from eight Dutch hospitals, specialized in AYA care, participated in semi-structured interviews. All interviews were audio-recorded, transcribed, and analyzed using a thematic analysis approach. Interviews with the healthcare providers revealed five themes concerning the discussion of sexual health: (1) being responsible for bringing up the topic of sexual health, (2) finding optimal timing to discuss sexual health, (3) acquiring knowledge to enable discussion of sexual health, (4) facilitating communication about sexual health, and (5) providing informative material for AYAs. To facilitate discussing sexual health, clear defined responsibilities within the team and sufficient knowledge are important. Self-report questionnaires, material to hand out, a checklist for healthcare providers, and knowledge of different communication strategies are helpful when discussing sexual health. By applying such means, communication with AYAs might be improved.

**Electronic supplementary material:**

The online version of this article (10.1007/s13187-020-01796-0) contains supplementary material, which is available to authorized users.

## Introduction

Sexual health is a multidimensional concept, the definition of which lacks consensus in the literature. For the purpose of this study, sexual health is comprised of sexual self-awareness, sexual function, and sexual relationships and intimacy [1–3]. These issues usually arise during adolescence or young adulthood [4, 5]. Cancer during this period may hinder normal sexual development as cancer and its treatment are associated with sexual problems [6–8]. Hence, there is a risk of a delay in sexual development in adolescent and young adults (AYAs; 15–39 years old) with cancer [9, 10]. As a result, AYAs are more likely to have impaired sexual function, decreased libido, and lower self-esteem [6]. Changes in body and body image are a major concern [11]. Issues with sexual health can have a negative impact on the development of intimate relationships which are of importance in coping with disease, and so it is necessary to pay attention to them in order to aid to psychological acceptance and recovery [9, 12, 13].

As well as desire for knowledge, AYAs have a need for support with the effect of cancer and its treatment on their sexual health. In a survey among 217 AYAs, 82% reported an unmet need regarding information and counselling on sexual health [14]. In addition, AYAs experience a lack of communication with healthcare providers [15]. In a retrospective study among 427 AYAs, only 12% had a discussion about sexual health within 6 months of the initial consultation [16]. AYAs’ discomfort and the presence of parents and family make it difficult for them to initiate a conversation themselves [15]. AYAs do, however, think that communication about sexual health with healthcare providers is important, as well as support in dealing with sexual problems, such as coping with physical side effects, issues around self-image, and learn to discuss their sexual concerns [11, 12, 17]. AYAs reported preferring to receive support in person from their healthcare providers [15, 17].

At the same time, sexual health is a challenging topic for healthcare providers to discuss with AYAs. There are numerous barriers that healthcare providers face, including lack of knowledge, lack of resources, low priority, presence of parents or family, their own discomfort, lack of time, and the lack of a longitudinal relationship with the patient [18–22]. Furthermore, there is a mismatch in expectations between AYAs and healthcare providers [20]. Although the majority of AYAs report an unmet need, in a survey accessing this aspect, only 28% of the oncology healthcare providers reported that AYAs’ need for sexual health went unmet [14].

Existing studies mainly focus on discussing fertility rather than sexual health, a topic which clinical teams need to address [11, 23–26]. As AYAs prefer a conversation in person about sexual health, it is important to know the practice and ideas of the healthcare providers in order to arrange realistic and feasible tools for them to enhance communication about sexual health. Therefore, with a view to improving sexual health care for AYAs, this study aims to gain insight into the views of healthcare providers on best practices.

## Methods

### Study Design

This is a qualitative investigation using semi-structured interviews with internist-oncologists, internist-hematologists, nurse specialists, and nurses specialized in AYA care regarding discussing sexual health [27]. This study was performed in collaboration with the Dutch “National AYA ‘Young and Cancer’ Network” (https://aya4net.nl/).

### Participants and Recruitment

There are eight academic hospitals with a specialization in AYA care in the Netherlands. Each of these hospitals has an AYA team consisting of an internist as team leader, specialized nurses, and allied healthcare providers. AYA care in the Netherlands is patient-centered and nurse-led [28]. The nurse and internist-oncologists or internist-hematologists are the first contact an AYA has when diagnosed with cancer. Therefore, this study included nurses and internist-oncologists or internist-hematologists, but no allied healthcare providers. In this paper, we describe them as “doctors” and “nurses,” lumped together as healthcare providers. The doctors and nurses of each of the eight AYA teams were invited to participate by e-mail. Healthcare providers with at least 1-year work experience with AYAs were eligible for this study. Fourteen healthcare providers agreed to participate in the study. Reasons for invited healthcare providers to decline the invitation were participation in other studies and lack of time. The participants who agreed to participate were contacted by phone to make an appointment for the interview.

### Data Collection

All interviews were performed by one of the authors (FB). As a medical student, she had followed trainings in interview techniques. Two test interviews were performed and evaluated to optimize technique applied. She had no connection with the participants prior to the interviews. In total, 13 interviews were conducted. Twelve of the participants were interviewed individually; one duo interview was performed as requested by two nurses. All interviews were conducted in person at the workplace of the interviewee, chosen by the interviewee. The duration of the interviews was approximately 1 h. After every two (new) interviews, the interviews were read and coded by two authors (FB and LA) and evaluated with regard to technique and quality. The set of questions set did not change. However, the interviewer focused more on facilitators for discussing sexual health than the exact current practice of the participants.

Supplementary Table S1 presents the semi-structured interview question set. The set is based on expert knowledge and literature investigating communication about sexual health in healthcare. Prior to the start of each interview, demographic information was checked by e-mail, including job, gender, age, number of years of experience in practice with AYAs, number of AYA patients seen per year, both in- and outpatient care, training in sexual health, and use of guidelines. Interviews were audio-recorded and transcribed verbatim. After each interview, the interviewer wrote a memo about the ambiance of the interviews and the themes discussed.

### Data Analysis

A thematic analysis was used to analyze the data [29]. First, transcripts were read thoroughly so that the two study authors (LA and FB) became familiar with the data; they were then coded independently. Discrepancies in coding were discussed with a third author (HE) until consensus was reached. Coding was supported by software program Atlas.ti.; 129 codes were defined. The codes were categorized into groups and later different themes were defined. Thematic saturation was reached, i.e., no new themes were mentioned by the interviewees, after the 10th interview. This indicated that this sample of 14 healthcare providers was adequate for capturing a range of responses. To be sure that no new topics were mentioned, three interviews continued after the 10th interview.

### Ethical Considerations

This study, the aim of which was to optimize the care process, was conducted among healthcare providers who participated voluntarily. After consultation with the Medical Ethics Committee of the LUMC, this study was considered as exempt from requiring ethical approval.

## Results

### Participants Characteristics

The sample included six doctors and eight nurses (Table 1). Seventy-nine percent (*n* = 11) were female. Mean age of the participants was 48 years. Participants came from seven (out of eight) different hospitals in the Netherlands. Two nurses and one doctor had followed a training on sexology. Two of the participants used guidelines to inform patients about sexual side effects of treatment.

**Table 1:** Demographics of participants (*n* = 14)

Characteristics	*N*
Function	
Internist: oncologist / hematologist	5 / 1
Nurse: specialists	8
Identified gender	
Female	11
Male	3
Age of participant in years	
30–40	3
41–50	5
51–60	5
61–70	1
Years of experience in practice with AYAs	
1–5	2
6–10	3
11–15	2
16–20	0
> 20	7
No. of contacts with AYA patients per year	
10–100	5
101–200	1
201–300	3
301–400	0
> 400	5

### Themes

“Being responsible for facilitating patients’ needs regarding sexual health” was defined as the overall theme of our study. The following five major themes were identified: (1) being responsible for bringing up the topic of sexual health, (2) finding optimal timing to discuss sexual health, (3) acquiring optimal knowledge to enable discussion of sexual health, (4) facilitating communication about sexual health, (5) providing informative material for AYAs.

#### Theme 1: Being Responsible for Bringing Up the Topic of Sexual Health

All participants emphasized the importance of discussing sexual health with AYAs. Some doctors described discussing sexual health as a role for nurses. They assumed that nurses have more time to discuss the topic and patients might experience a lower barrier talking to nurses. Some doctors noted discussing sexual health as being a role for themselves as well. They assumed they had a better doctor-patient relationship due to the frequent number of contacts they have with the AYAs. Some nurses stated that it was their responsibility because they see the patients more often; they assumed that patients experience a lower barrier to talking about sexual health and reported having time to discuss the topic. Others felt discussing sexual health was a shared responsibility and some stated that it does not matter who talked about it as long as the topic was discussed. A doctor highlighted the importance of clearly defined roles within the medical team regarding discussing this theme. He explained: “It should be clear who is responsible for discussing sexual health, otherwise it will not happen” (respondent 008).

#### Theme 2: Finding Optimal Timing to Discuss Sexual Health

Participants denoted different ideal moments for discussing sexual health during the treatment process. All participants stated that there is not one ideal moment to discuss sexual health. Some participants preferred at least to address the topic when the initial treatment plan is discussed. They consider it important to create awareness about this topic before starting treatment, by addressing possible sexual side effects. Some doctors argued to avoid this at the first appointment. They believe that the time available for giving a comprehensive explanation about the treatment itself is already limited. Sexual health does not form a priority at that moment. One stated: “Patients remember approximately 20% of a conversation; the more you discuss, the less they remember. I discuss more urgent side-effects first, like fever after chemotherapy” (respondent 003). Some healthcare providers preferred discussing sexual health during or at the end of treatment, in order to address possible sexual concerns directly, or opted for making a new appointment. “During the treatment process, it might be useful to make an extra appointment to discuss unsolved subjects, like sexual health”(respondent 003). Some healthcare providers described discussing sexual health only when there was a high chance of sexual problems, for instance after pelvic surgery. Eight healthcare providers mentioned the power of repetition; they consider it insufficient to discuss it only once.

#### Theme 3: Acquiring Knowledge to Enable Discussion of Sexual Health

Participants identified two major facilitators for achieving sufficient knowledge to discuss sexual health with the AYA: (1) education of the healthcare provider and (2) multidisciplinary approach.

Most healthcare providers did not receive education or training in sexology; according to them, this could be a facilitator for discussing the topic. They suggested that education during medical and nursing school would be ideal. “If the threshold for talking about sexual health is already lowered by education, it will be easier to talk about this subject later in your career” (respondent 004). Additionally, training could be provided for oncologists and oncology nurses. Other healthcare providers mentioned that an elective way of education would be ideal; for example, oncosexology training as part of an oncology course or a congress. The training should contain at least basic knowledge on cancer and sexual health and practical information on how to start a conversation about sexual health.

Healthcare providers described a need for a multidisciplinary approach to sexual health–related problems since they experience lack of knowledge and lack of expertise. Initiating a conversation about sexual health would feel more comfortable if there is an opportunity to discuss patients’ problem in a multidisciplinary team meeting. Additionally, they consider it important to have the option to refer AYAs to a sexologist within their hospital. One explained: “When I notice a problem with sexual health or the AYA has a question about sexual health, I discuss this in a multidisciplinary meeting in order to give good advice to the AYA” (respondent 004).

#### Theme 4: Facilitating Communication about Sexual Health

Two subthemes were defined: (1) tools for facilitating communication and (2) communication strategies to facilitate discussing sexual health with AYAs during a consultation.

##### Subtheme 1: Tools for Facilitating Communication

Various useful tools were mentioned as being helpful for improving communication about sexual health: (1.1) the use of a self-report questionnaire to complement the consultation, (1.2) informative material to hand, out and (1.3) a checklist for healthcare providers in the electronic patient dossier.

Most healthcare providers suggested the use of a self-report questionnaire which could be filled out by the AYA before the consultation with a doctor or nurse. (Both doctors and nurses conduct consultations.) It could include questions about sexual health in order to explore specific problems. One explained: “The AYA will be triggered to think about the subject and the healthcare provider will be more alert to discussing the topic” (respondent 003). Using the self-report questionnaire, the healthcare provider can prepare specific topics and it may lower the threshold for initiating a discussion about sexual health.

All healthcare providers also identified the availability of information material about sexual health as a facilitating tool. They consider it as helpful to be able to give some form of written information to the AYA. Furthermore, a checklist in the electronic patient dossier about whether sexual health is discussed was highlighted by several healthcare providers as a useful tool. They stated it would be helpful to become more aware of the topic. “AYAs meet multiple doctors and nurses. A checklist would be helpful to record if and when sexual health is discussed” (respondent 009).

##### Subtheme 2: Communication Strategies to Facilitate Discussing Sexual Health with AYAs During a Consultation

Healthcare providers described different communication strategies to facilitate discussing sexual health with AYAs in the consulting room. Six different strategies were identified: (2.1) actively initiating a discussion, (2.2) finding the right moment, (2.3) normalization of the subject, (2.4) actively returning to the subject, (2.5) use of humor, and (2.6) ensuring privacy.

### Actively Initiating a Discussion

One of the strategies which was frequently mentioned was to actively initiate a discussing of sexual health, instead of waiting for the AYA to bring up the topic. When the healthcare provider initiates the topic, it might be easier for AYAs to share their concerns. “Sometimes, AYAs dare not ask these questions” (respondent 001). Initiating the topic in response to another related topic, like fertility, was also recommended. “You actually need some triggers to start a discussion about sexual health. For example, hormone levels is a good topic for making it (sexual health) discussable” (respondent 008) or “Fertility is linked to sexual health. It is easier to start about sexual health when discussing fertility” (respondent 011).

### Finding the Right Moment

Some participants stated they preferred addressing sexual health in reaction to a question from the AYA. “I don’t want to meddle, I react to patients’ questions” (respondent 004). A question from the AYA can be used as an opening to explain more about sexual health and to go into more detail. “Sometimes patients themselves come along with a specific questions (about sexual health). That gives me the opportunity to discuss the broader topic” (respondent 002).

### Normalization of the Subject

Participants considered it important to normalize the subject. They explained: “It would be helpful if we normalize sexual health and ask about it just like asking about diet or weight” (respondent 002). Another way of normalizing the topic could be to emphasize that sexual health concerns are common. “You can tell the AYA that it is quite common to have issues with sexual health and that they are not an exception” (respondent 003).

### Actively Returning to the Subject

Some participants described actively returning to the topic of sexual health after the subject has been discussed in order to evaluate concerns. “When I advise the AYA about sexual health concerns, I always come back to the topic the next time. I check whether the advice worked well” (respondent 004).

### Use of Humor

Some of the healthcare providers suggested the use of humor as a strategy to bring up the topic of sexual health. “A bit of humor helps to bring a delicate subject like sexual problems out into the open” (respondent 012). Another explained: “Humor and sexuality are a good combination. It is a way of making the topic less loaded” (respondent 004).

### Ensuring Privacy

For an open discussion about sexual health, participants recommended ensuring having time alone with the AYA, without parents being present. “There is a world of difference between what AYAs tell if the parents are absent. The AYAs tend to ask more question regarding sexual health when they are alone” (respondent 005).

#### Theme 5: Providing Informative Material for AYAs

Participants were asked which elements should definitely be included in content of ideal information about sexual health for AYAs based on questions they receive from AYAs and items they consider to be important. Most healthcare providers stated that possible consequences and side effects of the cancer and its treatment should be included. Specific complaints per cancer type and treatment should be incorporated. One explained: “If the information is too general, they may again have doubts: does this also apply to me?” (respondent 001). Healthcare providers considered it important to include physiology of the healthy body, regarding sexual health, into the informative material. In this way, it is assumed that the effect of cancer on sexual health is easier to understand. Also the possible consequences for the partner, the influence on the relationship, and the influence on dating were mentioned here.

Some healthcare providers mentioned including the dos and don’ts regarding sexual health for AYAs. They notice AYAs being confused about whether or not it is allowed to kiss or have sex during chemotherapy or when to use a condom. It was also considered important to explain the rationale behind these statements in order to prevent possible “fables.” One healthcare provider explained: “There are a lot of fables about what is and isn’t allowed. I think healthcare providers caused this, because we give instructions without explaining the reason why” (respondent 003).

Moreover, practical tips in managing the side effects of the cancer and treatment were highlighted by several healthcare providers. As well as tips for physical and physiological management, tips for dating and to initiate a discussion on sexual health with their partner or healthcare provider were considered important.

Most participants agreed that information in the form of educational material about sexual health should be offered to the AYA. It is considered important that the material is accessible to every patient and healthcare provider and that the information about sexual health has a place among other subjects. Digital information via a website was considered by the majority of the healthcare providers to be the ideal form. Some participants also described an app or a podcast as a good online form. “A podcast could be very helpful to listen for listening to experiences of other patients and healthcare providers” (respondent 007).

Some healthcare providers described experiences of other AYAs to be a necessary element of the information material for AYAs. One doctor also mentioned the power of a film, in which AYAs recognize their own disease process. Finally, reliable sources for any additional information about sexual health and resources for specialized support (sexologist, psychologist, relationship therapist) were identified as useful in the attempt to provide the ideal information package.

## Discussion

In this study, we aimed to gain insight into perspectives and best practices of healthcare providers with regard to ideal way of discussing sexual health with AYAs, and their view on providing written information. According to most healthcare providers, discussing sexual health is a shared responsibility of doctors and nurses. There was, however, no overall consensus on the preferred timing. Participants highlighted the importance of offering information at various times and suggested education for the healthcare providers and multidisciplinary approach and communication tools as facilitating factors to enhance the discussion of sexual health with the AYA. A self-report questionnaire for the patients, material to hand out, and a checklist for healthcare providers were mentioned as facilitating tools. According to the participants, information material should at least be available online and include personalized information about sexual side effects, dos and don’ts and practical tips.

In concordance with earlier literature on adult oncology, participants in the current study allocated the responsibility for discussing sexual health to both nurses and doctors [19, 30, 31]. The importance of clearly defined roles within the medical team was highlighted. A qualitative study conducted by de Vocht et al. slightly supports this by showing that a complementary team approach, with clearly defined roles for different team members, is required to improve communication about sexual health in cancer and palliative care. In this model, some members of the oncology team, most likely the oncologist, discuss the sexual side effects of treatment and check whether patients need help during treatment to identify problems. Other members, most probably nurses, with some affinity with sexual health, have the role of supporting patients with their sexual health issues. Additional training and education could be provided to these members to improve their expertise [3].

In the current study, opinions differed about the optimal timing for discussing sexual health. Some participants preferred to at least address the topic when the initial treatment plan was discussed. We did not find literature about the opinion of AYA healthcare providers or of the AYAs themselves on best timing. It is known to be preference for discussing fertility at the start of treatment [18]. AYAs reported limited conversations with healthcare providers about sexual health [15]. An online questionnaire study among 667 female breast cancer (ex)patients emphasized the importance of appropriate timing for providing information about sexual health; these patients preferred to at least receive the information shortly after the treatment. Similar to our study, the importance of repetition of the information was mentioned [32]. This is also emphasized by AYAs, as they point out the difficulty in remembering the content of the conversations at the start of treatment and their request for ongoing communication throughout treatment and survivorship [15]. However, if the impact of treatment on sexual health is considered to be a side effect of treatment, this should possibly be explained when requiring informed consent. Informed consent is seen as an important part of medical practice and patients’ autonomy (23). Lack of clarity about sexual side effects in existing guidelines may result in ambiguity regarding responsibility and timing for discussing sexual health [3].

Sexual health is known to be an important but difficult topic to discuss. In our study, some facilitating factors to enhance the discussion have been described. First, in concordance with existing literature, participants emphasized the need for education for healthcare providers [15, 18]. This might contain basic and practical knowledge on sexual health implemented in an early stage of an oncologist’s or nurse’s training [18]. It would also help healthcare providers to overcome frequently reported barriers, such as lack of knowledge and discomfort and thus falling in line with the AYAs’ reported need for information about potential sexual side effects [15, 18]. A guideline for healthcare providers about sexual side effects in AYAs would also be useful. Participants in this study describe that it would be easier to initiate the conversation when having a multidisciplinary team meeting about sexual health issues and adequate referral options within their hospital. This is important as AYAs look for support and think the healthcare providers should initiate the conversation about sexual health [15].

Driven by the argument of discomfort, both the AYA and healthcare provider prefer this sensitive topic to be discussed without parents or family. AYAs suggested routinely asking parents or family to step out of the room for the sensitive part of the visits to the clinic [15, 18]. Some participants in our study suggested the use of humor as a strategy for rising the topic of sexual health. One should, however, be careful as the use of humor can be perceived as facilitating a discussion but also as derailing.

It is known that in cancer care, the request made by each AYA differs; this is also true for sexual healthcare. Therefore, it is important to adjust the care to the individual patient [15]. A self-report questionnaire, mentioned in the current study as a tool, might be helpful in clarifying individual needs of the AYA, and thus recognize problems at an early stage. Since conversation time is limited and sometimes AYAs do not dare initiate the subject, this might be a helpful tool [18]. Furthermore, the importance of addressing sexual problems as a matter of routine in AYA cancer care has been reported [11].

As well as face-to-face conversations with healthcare providers, AYAs report a need for resources [15, 18]. Healthcare providers in our study contributed some ideas about content and form of information provision. According to them, the material should at least be adjusted to the type and stage of different forms of cancer, so that it becomes more patient-centered.

### Limitations

This study was a nationwide survey in collaboration with the national AYA healthcare network. It does have certain limitations. First of all, the sample size was small and most participants were female. However, data saturation was reached with our number of participants and the male-female ratio is a reflection of the ratio in the Netherlands. Secondly, allied healthcare providers like sexologists were not included; this might affect the results. In the Netherlands, AYAs do not have an appointment with a sexologist as a matter of routine. Only those AYAs with sex-related problems, if recognized as such, will be referred to allied healthcare providers. In future studies, it might be relevant to explore the perspective of sexologists and psychologist to formulate more best practices for discussing sexual health. Finally, selection bias may have occurred. Healthcare providers who agreed to participate in this study may have more affinity with this subject. It could be that the perspective of the healthcare provider with less affinity for this subject is underexposed. However, as described before, it is more important to allocate responsibility for discussing sexual health within the team rather than expecting everyone to address the topic in depth.

## Conclusion

Cancer can interfere with sexual development of the AYA and may cause problems with sexual health. Sexual health is an important quality-of-life concern; problems with sexual health may negatively affect the quality of life of AYAs [7, 11]. This study described the view of the healthcare provider on best practices to meet AYAs’ needs regarding sexual health. To facilitate discussing sexual health, clearly defined responsibilities within the team are important. Additionally, sufficient education and the opportunity to discuss sexual concerns in a multidisciplinary meeting are major facilitators for enhancing healthcare providers’ knowledge. Self-report questionnaires for the patients, material to hand out, and a checklist for healthcare providers could be helpful tools to facilitate discussion. Communication strategies for discussing sexual health are actively initiating a discussion, finding the right moment, normalization of the subject, actively returning to the subject, careful use of humor, and ensuring privacy. After all, participation of the AYAs in prioritizing their own care according to their needs for sexual health is crucial.

## Electronic Supplementary Material

ESM 1 (DOCX 15 kb)

## Data Availability

Available on request (in Dutch).
