# Supplementary material for: Discussing Sexual Health with Adolescent and Young Adults with Cancer: a Qualitative Study Among Healthcare Providers
Source: J Cancer Educ. 2020 Jun 17;37(1):133–40. doi: 10.1007/s13187-020-01796-0 (PMC8816785; doi:10.1007/s13187-020-01796-0)
Supplement: Supplementary file 1 — (DOCX 15 kb) [file 13187_2020_1796_MOESM1_ESM.docx]

**SUPPLEMENTARY**
**TABLE S1** Semi-structured interview questions

| **Number** | **Question** |
| --- | --- |
| 1 | I would like to begin this interview by asking you to describe the type of verbal information your practice currently provides on sexual health for AYA patients. |
| 2 | With regard to verbal information on sexual health, what should this be in your opinion? |
| 3 | What gaps or barriers do you currently notice in the verbal information provided? How could these be solved? |
| 4 | Barriers in communication about intimacy and sexuality are also exposed in the literature; for instance: lack of knowledge about/experience in having the conversation, lack of resources/referrals for patients, low priority, presence of parents/family, patient discomfort, clinician discomfort, time and a lack of rapport/longitudinal relationship. How could this be solved? |
| 5 | Who should discuss sexual health with AYAs? |
| 6 | When should sexual health be discussed with AYAs? |
| 7 | In terms of content, what aspects of sexual health should be discussed with AYAs? |
| 8 | If your practice provides written information on sexual health for AYA patients, what form does this take? |
| 9 | Do you feel a certain need for informative material about sexual health for AYAs? |
| 10 | In your opinion, what should informative material on sexual health for AYAs be? |
| 11 | In terms of content, what should the written information on sexual health for AYAs contain? |
